# A Case of Nonimmune Hydrops Fetalis With a Duct‐Dependent Systemic Circulation and a Novel Mutation of Kabuki Syndrome

**DOI:** 10.1155/crig/8898702

**Published:** 2026-05-29

**Authors:** Rameshwar Prasad, Sudipta Sahoo, Richie Dalai, Keshav Kumar Pathak, Bhabesh Kant Chowdhary

**Affiliations:** ^1^ Department of Neonatology, All India Institute of Medical Sciences, Patna, Bihar, India, aiims.edu

**Keywords:** Kabuki syndrome, neonatal intensive care, nonimmune hydrops

## Abstract

**Introduction:**

Nonimmune hydrops fetalis (NIHF) has numerous etiologies, the most common of which are cardiac anomalies and fetal infection. However, genetic disorders are also being increasingly recognized as a cause of NIHF. Here, we report a case of a neonate presenting with polyhydramnios, NIHF, structural heart disease, and diaphragmatic defect who was found to have a previously unreported mutation in the *KMT2D* gene.

**Case Presentation:**

A female neonate with antenatally detected NIHF was born at 35 weeks of gestation via cesarean section. At birth, she was noted to have dysmorphic features, scoliosis, and a single umbilical artery. Further investigations revealed a left‐sided obstructive cardiac lesion and a right‐sided Morgagni hernia. She required invasive ventilation, inotropes, and prostaglandin E1 for preductal coarctation of the aorta with hypoplastic left heart syndrome. Genetic analysis was warranted due to multiple anomalies in the neonate. Whole exome sequencing (WES) showed a previously unreported truncating mutation in the *KM2TD* gene, confirming the diagnosis of Kabuki syndrome type 1.

**Conclusion:**

Kabuki syndrome is rare, and its presentation with hydrops is extremely rare. Our case presented with polyhydramnios, antenatal hydrops, hypoplastic left heart, right‐sided Morgagni hernia, and scoliosis with a novel mutation, thus potentially expanding the genotype–phenotype spectrum of this syndrome. This case highlights that a pediatrician should have a high index of suspicion for inherited genetic syndromes in a case of nonimmune hydrops with multiple congenital anomalies. Genetic tests are valuable for identifying rare syndromes and novel mutations.

**Summary**
• What is already known?
◦ Nonimmune hydrops fetalis (NIHF) in neonates is multifactorial.
◦ Kabuki syndrome is a rare syndrome, and its presentation with nonimmune hydrops is extremely rare.
• What does this study add?
◦ In the cases of nonimmune hydrops fetalis (NIHF), a thorough antenatal and postnatal evaluation for the delineation of major congenital anomalies is essential for the management of NIHF.
◦ Genetic analysis in the index case showed a previously unreported truncating mutation in the *KM2TD* gene and expands the genotype–phenotype spectrum of Kabuki syndrome.

## 1. Introduction

Nonimmune hydrops fetalis (NIHF), characterized by the presence of abnormal fluid collections at ≥ 2 sites in the absence of red cell alloimmunization, is caused by numerous disorders. Cardiac anomalies and fetal infection are among the most common etiologies [1]. Genetic syndromes are also being increasingly recognized in cases of NIHF. One of these is Kabuki syndrome (KS), which has an estimated incidence of 1 in 32,000 births. It was first independently described by Niikawa et al. and Kuroki et al. [2, 3] The five cardinal features of KS include dysmorphic facies, short stature, intellectual disability, skeletal anomalies, and characteristic dermatoglyphics, as described by Niikawa et al. [2] The characteristic facies is long palpebral fissures with eversion of the lateral third of the lower eyelids, which resembles the facial features of the actors of Kabuki, a form of traditional theater in Japan.

KS is caused by a mutation in the *KMT2D* (KS type 1) and *KDM6A* (KS type 2) genes, located on chromosome 12 and X, respectively. These genes are involved in extensive epigenetic modification of gene expression, accounting for the multisystem involvement characteristic of this disease. Genetic analysis techniques aid early diagnosis, parental genetic counseling, and prognostication. In this case report, we present a neonate with NIHF, structural heart disease in the form of a duct‐dependent systemic circulation, and a right‐sided diaphragmatic defect with a novel variant in the *KMT2D* gene. This case presents a previously unreported mutation for KS, which is a rare syndrome, and a presentation as NIHF, which is even rarer. To the best of our knowledge, these combinations of findings have not yet been reported in KS.

## 2. Case Presentation

### 2.1. The Initial Presentation

A 29‐year‐old woman, third gravida with one prior abortion due to an unknown cause and one healthy living issue, was referred to the tertiary care neonatal intensive care unit (NICU) at the All India Institute of Medical Sciences, Patna, Bihar, India, in May 2024, at 35 weeks of gestation. The conception in her current pregnancy was nonconsanguineous and spontaneous, and her initial antenatal period was uncomplicated. Ultrasonograms (USGs) at the 8^th^ and 17^th^ weeks of gestation were normal. However, at 34 weeks of gestation, her USG revealed the presence of polyhydramnios, mild pleural effusion, and ascites in the fetus, following which she was referred to us. These findings were confirmed with a repeat USG at our center. There was no setting of Rh‐isoimmunization, and MCA‐PSV was normal. Antenatal evaluation done for NIHF included a fetal echocardiogram, which was reported normal. Investigations for viral markers and other intrauterine infections were negative. A full course of antenatal corticosteroids was given before delivery by cesarean section, and a female neonate weighing 2110 g was born.

At birth, the neonate required resuscitation with positive pressure ventilation. She had Apgar scores of 4, 7, and 9 at 1, 5, and 10 min of life, respectively. Thereafter, due to persistent respiratory distress after birth, she was kept on continuous positive airway pressure (CPAP) and transferred to the NICU. In the NICU, she was kept on noninvasive positive‐pressure respiratory support (NIPPV) due to persistent respiratory distress, with initial settings of peak inspiratory pressure (PIP) 15, positive end‐expiratory pressure (PEEP) 5, and a fraction of inspired oxygen (FiO2) 30%.

**FIGURE 1:**
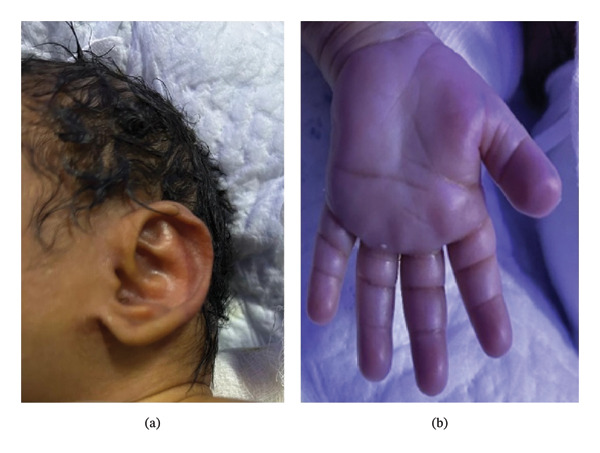
Dysmorphic features in the index neonate with Kabuki syndrome. (a) Auricular dysmorphism in the form of a large cupped‐shaped outer ear and (b) changes in dermatoglyphics in the form of a persistent fetal fingertip pad and brachydactyly.

On examination, she was found to be hypotonic, had arched eyebrows with sparse hair, large, cupped ears, persistent fetal fingertip pads, broad, short hands, brachydactyly, thick nape of the neck, single umbilical artery, and scoliosis (shown in Figure 1). The neonate had skin edema and ascites. As the neonate presented with hydrops and dysmorphic features, further workup was done to rule out major congenital anomalies.

### 2.2. The Management and Outcome

**FIGURE 2:**
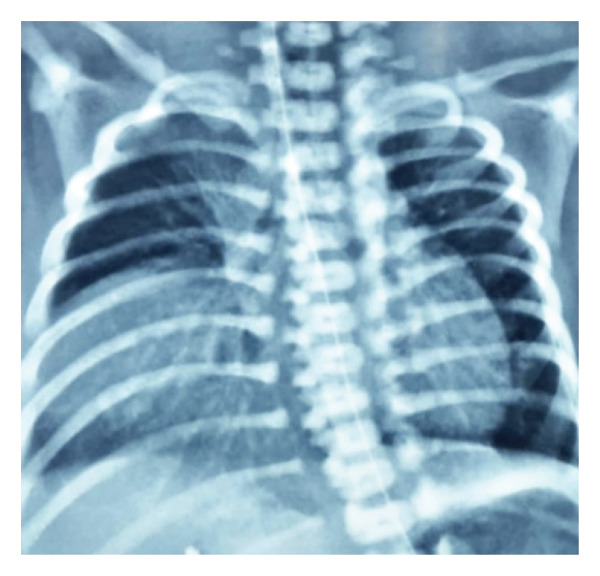
A thoracic radiogram with anteroposterior view showing a well‐circumscribed opacity in the right lower pulmonary zone and the paracardiac region.

A bedside echocardiogram done on Day 2 of life showed mild pericardial effusion and atrioventricular canal defect. However, due to a poor acoustic window, further evaluation of cardiac and extracardiac structures could not be done, and a computed tomography (CT) angiography was planned for the accurate depiction of complete cardiovascular morphology. Other ancillary tests for the etiology of hydrops were also done, which showed both the mother’s and neonate’s blood groups as O positive, a negative direct Coombs’ test, and a normal complete blood count. Maternal indirect Coombs test and serologic test for syphilis and antibodies for toxoplasma, CMV, and rubella were also negative. As the neonate had respiratory distress, a chest radiograph showed a well‐circumscribed right lung opacity (shown in Figure 2). The possibilities considered were congenital pneumonia, pulmonary sequestration, congenital pulmonary airway malformation, right‐sided eventration, or defect of the diaphragm. The respiratory distress in the neonate gradually improved, and she was shifted to CPAP support on Day 4 of life. The CT angiogram done revealed the presence of a hypoplastic left heart, preductal coarctation of the aorta, aberrant right subclavian artery, a very narrow patent ductus arteriosus, no significant aortopulmonary collaterals, and a right‐sided Morgagni hernia with liver as content. The neonate subsequently developed signs of shock with pallor, cold peripheries, and feeble pulses for which she was started on prostaglandin E1 (PGE1) infusion, was intubated, and put on mechanical ventilation. The shock improved following the PGE1 infusion.

Given the multitude of malformations, nonimmune hydrops, and a suspicion of a genetic syndrome, whole exome sequencing (WES) was conducted. It revealed a heterozygous nonsense variant (NM_003482.4: c.9979C > T) in exon 35 of the *KMT2D* gene (chr12:g.49037377G > A; Depth:148×) that resulted in a stop codon and premature truncation of the protein at codon 3327 (p.Gln3327Ter; ENST00000301067.12). This variant has not been reported in the 1000 genomes, gnomAD (v3.1), gnomAD (v2.1), topmed, or in internal databases. The *in-silico* prediction of the variant was damaging by MutationTaster2 (MT2). This variant fits the PS1 (null variant with loss of function in gene) and PM2 (absent from controls) categories according to the American College of Medical Genetics and Genomics 2015 guideline and was classified as likely pathogenic [4]. The sequences obtained were aligned to the GRCh38 human reference genome. The Matched Annotation from National Center for Biotechnology Information (NCBI) and the European Molecular Biology Laboratory’s European Bioinformatics Institute (EMBL‐EBI)‐the “MANE Select” transcript was used for clinical reporting. MT2 predictions are based on NCBI/Ensembl 66 build (GRCh38 genomic coordinates are converted to hg19 using University of California, Santa Cruz (UCSC) LiftOver, and mapped to MT2). The existing population allele frequencies (1000Genome and gnomAD‐Exome) are currently available for the hg19 genome version only.

For hypoplastic left heart syndrome and Morgagni hernia, cardiothoracic and pediatric surgery consultation was sought. However, the parents refused any surgical intervention and opted for palliative care due to multiple malformations. The neonate succumbed to the underlying malformations at 1 month of life. The timeline of events has been elucidated in Table 1.

**TABLE 1:** Timeline of events.

Day of life (DOL)	System/domain	Event/intervention	Key findings/outcome
DOL 1‐2	Birth and resuscitation	Late preterm female infant born	BW 2.11 kg, Apgar 4/7/9 PPV for 2 min
	Respiratory	Started on CPAP, followed by NIPPV, and shifted to the NICU	SAS score 4/10
	Imaging	USG thorax and abdomen	Mild pericardial and pleural effusion, ascites
	Nutrition	Minimal enteral feeds	Feeds tolerated

DOL 2–4	Respiratory	Shifted to CPAP	
	Sepsis	Antibiotics started	Piperacillin tazobactam + Amikacin
	Nervous system	USG cranium	Normal
	Nutrition	Full feeds achieved	By 48 h

DOL 5 DOL 14–30	Cardiovascular	CT chest with pulmonary angiography	HLHS with coarctation, PDA, Duct‐dependent circulation, PGE1 infusion started
	Respiratory	Mechanical ventilation	Electively intubated due to worsening distress
	Palliative care	Parents opted for palliative care due to the overall poor prognosis	Whole exome sequencing done

*Note:* NIPPV: noninvasive positive pressure ventilation; USG: ultrasonogram; PGE1: prostaglandin E1.

Abbreviations: CPAP = continuous positive airway pressure, CT = computed tomogram, HLHS = hypoplastic left heart syndrome, NICU = neonatal intensive care unit, SAS = Silvermann–Anderson Score.

## 3. Discussion

The presence of dysmorphism and multiple congenital anomalies, in addition to Morgagni hernia in the index case, prompted us to search for syndromic associations of NIHF. A homogenous opacity on chest radiograph, on further evaluation with CT thorax, revealed a right‐sided diaphragmatic hernia. Although bedside echocardiography is a valuable tool for the assessment of congenital heart diseases, in our case, the poor acoustic window due to altered cardiac position resulting from scoliosis and right diaphragmatic hernia hindered the timely diagnosis of a duct‐dependent systemic circulation, and the final diagnosis was confirmed only with a CT. Due to the presence of a critical congenital heart disease, diaphragmatic hernia, fetal hydrops, and dysmorphic features, the possibilities of trisomy, Fryn syndrome, and KS were kept. However, the WES established the final diagnosis as KS.

A diagnosis of KS is made in a neonate of any gender and any age with hypotonia, developmental or intellectual disability, and the presence of either of the two major criteria, i.e., mutation in the *KMT2D* or *KDM6A* gene and typical dysmorphic features [5]. The typical facial features of long palpebral fissures and eversion of the lateral third of the lower eyelid were absent in our case. Neonatal presentation of KS is challenging to diagnose, owing to the variable manifestations. In addition, not all typical facial features are evident in neonates.

KS with right‐sided Morgagni hernia and postductal coarctation has been reported previously. However, there was no polyhydramnios or hydrops fetalis in that case [6]. Cases of KS with hydrops and left‐sided cardiac defects have rarely been reported [7, 8]. The clinical presentation of KS is heterogeneous, with a combination of cardiac defects, polyhydramnios, genitourinary anomalies, single umbilical artery, hydrops, and pleural and pericardial effusion reported in different cases [9]. Of these, cardiac abnormalities are among the most frequent malformations; left‐sided obstructive lesions are the most common type, reported in KS [10, 11]. In one case series, 14/15 cases had cardiovascular malformations. However, in seven of them, cardiovascular malformations were not detected in antenatal ultrasound [12]. Polyhydramnios has been reported in 25%–41% pregnancies in KS [13, 14]. Genitourinary anomalies and a single umbilical artery are the other commonly reported anomalies in antenatal ultrasound in KS [14]. A review of all published confirmed cases of KS revealed that the frequency of genitourinary anomalies and single umbilical artery were 26.5% and 15.7%, respectively [9]. Nonimmune hydrops is a less frequent presentation of KS occurring in 12% of confirmed cases [9, 12, 13]. Most cases of nonimmune hydrops in KS were associated with the *KMT2D* gene mutation [15]. Diaphragmatic defects have been infrequently reported in KS, typically occurring on the right side and often associated with Morgagni hernia [16].

Most cases of KS are caused by an autosomal dominant *de novo* mutation in the *KMT2D* gene. The *KMT2D* variants are truncation mutations in most KS patients, as was the case in our study. However, the mutation found in the index case (a heterozygous nonsense variant in exon 35 of the *KMT2D* gene (chr12:g.49037377G > A) that resulted in a stop codon and premature truncation of the protein at codon 3327 (p.Gln3327Ter) has not yet been reported in any public database. The likely pathogenic *KMT2D* variant and the phenotypic features of our case were consistent with a diagnosis of KS type 1 (OMIM#147920).

One of the major clinical challenges in this case has been the difficulty in diagnosing the nature of lung opacity and cardiac anomaly, as both conditions remained undetected in the prenatal ultrasound. An additional challenge in the case of NIHF was establishing the diagnosis and providing parental counseling. The patient exhibited dysmorphic features, with phenotypic characteristics overlapping previously reported findings, along with additional features, thereby expanding the clinical spectrum of KS1. The detection rate of fetal congenital anomalies with antenatal ultrasonography usually remains suboptimal in low‐ and middle‐income countries [17]. A report by Qin Y et al. on 6 cases of KS for characteristic features in prenatal ultrasound showed that the majority of the fetuses had cardiac (65.7%) and renal (57.1%) anomalies, while fetal hydrops was identified in 11.4% of the fetuses [18]. Another case of transient nonimmune hydrops was reported by Uzun Çilingir I et al., where the hydrops initially detected at 18 weeks disappeared by 32 weeks of gestation without any other major structural malformations [19]. In low‐ and middle‐income countries where proper antenatal follow‐up and availability of USGs are issues, there is a huge challenge for the timely management of such cases. Similarly, due to the lack of an antenatal diagnosis, this case posed a great challenge in front of us, and the diagnosis of the multisystemic malformations was resolved only after postnatal imaging studies. Genetic testing of parents could not be done due to a lack of consent. Hence, this limitation precludes the determination of whether the *KMT2D* variant arose *de novo* or was inherited. Parental testing enables us to establish the inheritance pattern and to detect possible parental mosaicism and, therefore, allows the accurate assessment of recurrence risk.

In conclusion, our case emphasizes the need for a thorough antenatal as well as postnatal evaluation for a case of NIHF, including fetal echocardiogram, and underscores the significance of a molecular confirmation. Left‐sided obstructive cardiac anomalies with a right‐sided diaphragmatic defect in such cases may be a clue toward KS. And as the eyes only see, what the mind already knows, training of healthcare professionals, especially in rural and suburban areas in antenatal and postnatal theragnostic for NIHF, will probably improve the survival of these neonates.

## Author Contributions

Rameshwar Prasad and Sudipta Sahoo prepared the initial manuscript. Richie Dalai, Keshav Kumar Pathak, and Bhabesh Kant Chowdhary added to the literature review and did a critical revision of the final manuscript.

## Funding

The authors have nothing to report.

## Disclosure

All authors gave their approval for the final version of the manuscript.

## Ethics Statement

As the study was a case report and patients’ anonymity was maintained, it was exempt from requiring ethics approval.

## Consent

The father of the neonate has provided written informed consent regarding the case presentation and publication of the medical details and images of the neonate in a deidentified manner.

## Conflicts of Interest

The authors declare no conflicts of interest.
